# Clinico-epidemiological characteristics of men and women with a new diagnosis of chronic obstructive pulmonary disease: a database (SIDIAP) study

**DOI:** 10.1186/s12890-021-01392-y

**Published:** 2021-01-28

**Authors:** Josep Montserrat-Capdevila, Josep Ramon Marsal, Marta Ortega, Maria Teresa Castañ-Abad, Miquel Alsedà, Ferran Barbé, Pere Godoy

**Affiliations:** 1 Unitat Docent Multiprofessional d’Atenció Familiar i Comunitària (UDMAFiC) Lleida-ICS, Gerència Territorial de Lleida ICS, Lleida, Catalonia Spain; 2 Atenció Primària, Institut Català de la Salut (ICS), Lleida, Catalonia Spain; 3 Faculty of Medicine, Universitat de Lleida (UdL), Lleida, Catalonia Spain; 4 Biomedical Research Institute of Lleida (IRBLleida), Lleida, Catalonia Spain; 5 Cardiovascular Epidemiology Unit, Hospital Universitari Vall d’Hebron, Barcelona, Catalonia Spain; 6 Research Support Unit, Direcció d’Atenció Primària ICS-Lleida; Institut Universitari d’Investigació en Atenció Primària (IDIAP Jordi Gol), Institut Català de la Salut, Lleida, Catalonia Spain; 7 Agència de Salut Pública de Catalunya, Departament de Salut, Lleida, Catalonia Spain; 8 Hospital Universitari Arnau de Vilanova, Lleida, Catalonia Spain; 9 Centro de Investigación Biomédica en Red de Enfermedades Respiratorias (CIBERES), Madrid, Spain; 10 Centro de Investigación Biomédica en Red de Epidemiología y Salud Pública (CIBERESP), Madrid, Spain

**Keywords:** Chronic obstructive pulmonary disease, Prevalence, Epidemiology

## Abstract

**Background:**

The risk of developing Chronic Obstructive Pulmonary Disease (COPD), the associated comorbidities and response to bronchodilators might differ in men and women. The objective of this study was to determine the prevalence of COPD and the clinic-epidemiological characteristics of primary care patients with COPD according to gender.

**Methods:**

This is a cross-sectional study using electronic healthcare records Catalonia (Spain), during the 01/01/2012–31/12/2017 period. Patients from the SIDIAP database (System for the Development of Research in Primary Care) were included (5,800,000 patients registered in 279 primary care health centres). Clinic-demographic characteristics, comorbidities and blood tests results were collected for each patient. Adjusted OR (ORa) with logistic regression methods were used to determine variables associated with men and women.

**Results:**

From an initial sample of 800,899 people, 24,135 (3%) were considered COPD patients, and 22.9%were women. The most common risk factors in women were bronchiectasis (ORa = 20.5, SD = 19.5–21.6), age > 71 years (ORa = 18.8; SD = 17.3–20.5), cor pulmonale (ORa = 5.2; SD = 4.3–6.7) and lung cancer (ORa = 3.6, SD = 3.2–4.0). Men and women presented the same comorbidities, though the strength of association was different for each gender.

**Conclusions:**

Patients suffering high comorbidity rates. Comorbidities are similar in men and women, although the strength of association varies according to gender. Women are more susceptible to the harmful effects of smoking and present a higher proportion of bronchiectasis and OSAS.

## Background

Chronic Obstructive Pulmonary Disease (COPD) is a condition which causes high morbidity and mortality globally [1], and is currently considered one of the main public health issues [2]. While WHO estimates that there are 251 million people affected with COPD globally [2], the EPISCAN study (Epidemiologic Study of COPD in Spain) reported that in Spain over a million and a half patients (73%) remained undiagnosed and untreated, and thus at higher risk of exacerbations and disease progression [2]. The main prevalence studies of COPD in the Spanish population are IBERPOC and EPISCAN. In 1997, IBERPOC showed a COPD prevalence of 9.1%, similar to the prevalence found a decade later by EPISCAN (10.2%) [3]. COPD prevalence is highly heterogeneous, and it varies according to geographical location, the sample used and the definition of the disease. A systematic review on the prevalence of COPD in Europe found prevalence ranging between 2.1 and 26.1% in people over 40 years of age [4]. In the Vallès region of Catalonia, the prevalence of COPD using the criteria of the British Thoracic Society was 7.2% (10.4% in men and 4.1% in women) [5]. A recent study conducted by our group in the population of Lleida (Catalonia) showed a prevalence of 14.5% (16.1% in men and 12.3% in women) [6].

COPD is associated with age, smoking [7] and cardiovascular diseases [8]. The risk associated with COPD might be similar to that of widely accepted cardiovascular factors such as hypertension and hypercholesterolaemia [8]. Various studies have pointed at the high prevalence of cardiovascular diseases, diabetes, kidney failure and other diseases associated with COPD [9].

Patients with COPD attend frequently primary care and emergency services for problems related to comorbidities and exacerbation [10]. Consequently, primary care patients can be considered a suitable sample of the general population. Some studies indicate that risk patterns might be different in men and women [11, 12], with different susceptibilities to develop COPD, associated comorbidities and response to bronchodilators [11]. Understanding gender differences in risk factors and comorbidities related to COPD might contribute to minimise their impact and improve the quality of life of these patients [13].

The objective of this study is to determine the prevalence of COPD and the clinico-epidemiological characteristics by gender in patients who attended primary care centres in Catalonia during the 2012–2017 period.

## Methods

Observational epidemiological study on the prevalence of COPD according to primary care consultations in Catalonia (Spain), with data originating from electronical medical records from 01/01/2012 to 31/12/2017. The study aimed to discriminate clinico-epidemiological characteristics between genders. Participants were eligible patients included in the SIDIAP (System for the Development of Research in Primary Care) [14], a database with anonymised information from 5,800,000 patients assigned to 279 primary care centres of the Catalan Health Institute (approximately 80% of the Catalan population). In Catalonia, health professionals use ICD-10 to classify diseases in the electronic medical records (eCAP).

### Population with a new diagnosis of COPD

**Fig. 1:**
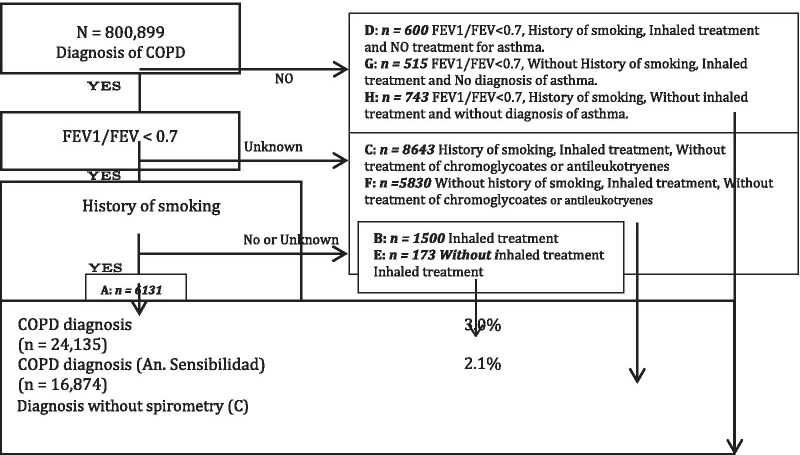
Flowchart of the study patients

Patients with COPD were identified for each year of the study period (2012–2017) using a diagnostic algorithm developed in previous studies that used similar databases [15]: Following GOLD [16] guidelines, patients over 40 years of age diagnosed with COPD, emphysema or chronic bronchitis and with FEV1/FVC < 0.7 were included to perform spirometry. Patients diagnosed with COPD with spirometry result unknown, who were smokers/ex-smokers and received bronchodilator therapy (excluding treatment with cromoglycate and antileukotrienes), or who had never been smokers but received bronchodilator therapy (excluding treatment with cromoglycate and antileukotrienes) were also included. Patients not diagnosed with COPD but with spirometry results of FEV1/FVC < 0.7, not diagnosed nor treated for asthma were also included (see Fig. 1.).

Information on clinico-demographic characteristics, including history of smoking and alcohol consumption, comorbidities, blood tests results, bronchodilator therapy and immunizations (influenza and pneumococcal) was obtained for each patient. Severity of COPD was evaluated in accordance to the most recent review of the 2018 GOLD Report [17].

The comorbidities (heart failure, ischemic heart disease, diabetes and prediabetes, chronic kidney disease, atrial fibrillation, anaemia, hypertension, dyslipidaemia, stroke and lung cancer) were obtained from the primary care electronic medical records and the hospital discharge minimum data set.

The study was approved by the Clinical Research Ethics Committee of the Institut d’Investigació en Atenció Primària Jordi Gol of Barcelona (P13/063).

### Statistical analysis

All data originated from the SIDIAP database. Patients were classified in two main groups (with and without characteristics suggestive of COPD) (Fig. 1). Data on the characteristics of the patients, diagnoses recorded by family doctors, and pharmacological treatment dispensed in pharmacies (in Spain, medicines are subsidized by the universal health care system) were collected. Variables were described with mean and standard deviation in case of continuous variables, and with absolute and relative frequencies in case of categorical variables. Differences between groups were analysed using Student’s *t*-test for continuous and Chi-squared test for categorical variables. Differences between groups were estimated calculating the difference of proportions and means. Logistic regression was used to estimate the crude and adjusted effects in both genders. The logistic regression models for men and women were built using backward elimination, with a significance threshold of *p* < 0.1.

## Results

A total of 24,135 (3%) out of the 800,899 people of the sample were considered to present clinical characteristics suggestive of COPD (Fig. 1); 22.9% were women, with an average age of 72 years (SD = 11). The highest proportion of patients with COPD was in 71 to 80 year age group (32.8%). The proportion of patients that were smokers or ex-smokers was 66.8% in patients diagnosed with COPD, compared to 31.5% in patients without COPD (*p* < 0.001). In relation to alcohol consumption, 39.8% of patients with COPD admitted to a low, moderate or high risk drinking, compared to 31.1% of patients with no COPD (*p* < 0.001). With respect to severity (GOLD), 49.1% patients with COPD were classified as moderate and 38.5% as severe. The average forced expiratory volume in one second (FEV1) in patients with COPD was 57.7 (SD = 19.8). Table 1 shows the remaining variables.

**Table 1:** Characteristics of SIDIAP patients at the beginning of the study

	No (n = 776,764; 97%)		Yes (n = 24,135; 3%)		Dif	Total (n = 800,899; 100%)		*p*
	N	n (%)	N	n (%)		N	n (%)	
Gender (female)	776,764	406,346 (52.3%)	24,135	5523 (22.9%)	−29.4%	800,899	411,869 (51.4%)	< 0.001
Age	776,764	60 ± 14	24,135	72 ± 11	12	800,899	60 ± 14	< 0.001
Grouped Age	782,055		18,844			800,899		< 0.001
41—50		254,121 (32.5%)		952 (5.1%)	−27.4%		255,073 (31.8%)	
51—60		192,339 (24.6%)		2964 (15.7%)	−8.9%		195,303 (24.4%)	
61—70		153,292 (19.6%)		5471 (29%)	9.4%		158,763 (19.8%)	
71—80		107,013 (13.7%)		6174 (32.8%)	19.1%		113,187 (14.1%)	
80 or more		75,290 (9.6%)		3283 (17.4%)	7.8%		78,573 (9.8%)	
Smoking History (Y)	776,764	244,600 (31.5%)	24,135	16,117 (66.8%)	35.3%	800,899	260,717 (32.6%)	< 0.001
Alcohol Consumption	371,525		17,843			389,368		< 0.001
No consumption		255,958 (68.9%)		10,734 (60.2%)	−8.7%		266,692 (68.5%)	
Low Risk		107,170 (28.8%)		6374 (35.7%)	6.9%		113,544 (29.2%)	
Moderate Risk		8301 (2.2%)		725 (4.1%)	1.8%		9026 (2.3%)	
High Risk		96 (0%)		10 (0.1%)	0.0%		106 (0%)	
Waist Circumference	66,004	101.6 ± 12.3	4183	105.8 ± 12.7	4.14	70,187	101.9 ± 12.4	< 0.001
Missing	776,764	710,760 (91.5%)	24,135	19,952 (82.7%)	−8.8%	800,899	730,712 (91.2%)	< 0.001
Grouped BMI	280,871		15,400			296,271		0.310
Normal		59,635 (21.2%)		3306 (21.5%)	0.2%		62,941 (21.2%)	
Overweight		120,728 (43%)		6451 (41.9%)	−1.1%		127,179 (42.9%)	
Obesity		100,508 (35.8%)		5643 (36.6%)	0.9%		106,151 (35.8%)	
Gold grouped			9746			NA		
I				1205 (12.4%)				
II				4788 (49.1%)				
III–IV				3753 (38.5%)				
Without bronchodilation								
FEV1/FVC			9213	59.6 ± 11.4				
FEV1			9054	57.7 ± 19.8				
FVC			8826	100.1 ± 201.6	FVC			8826

Table 2 shows comorbidities and blood tests results (fibrinogen and C-reactive protein in peripheral blood) of patients with and without a COPD diagnosis. Up to 26.7% of patients with COPD had a diagnosis of type 2 diabetes, 6.6% of prediabetes (capillary glycaemia between 110 and 125 mg/dL), 59.1% presented hypertension (HT) and 45.7% dyslipidaemia. Additionally, 13.1% had anaemia, 11.8% atrial fibrillation, 8.8% ischaemic cardiomyopathy, 11.7% chronic kidney disease, 9.4% heart failure, 8.1% had a history of stroke and 2.1% of lung cancer. Regarding blood tests results, mean fibrinogen in peripheral blood in patients with COPD was 407.2 mg/dL (SD = 105.7) and mean C-reactive protein (PCR) 15.3 (SD = 32.5). For all described comorbidities and blood tests results, statistically significant differences were found in COPD versus non COPD patients.

**Table 2:** Comorbidities and blood tests results in patients with and without COPD

COPD	No (n = 776,764; 97%)		Yes (n = 24,135; 3%)		Dif	Total (n = 800,899; 100%)		*p*
	N	n (%)	N	n (%)		N	n (%)	
DM2 (Y)	776,764	98,932 (12.7%)	24,135	6453 (26.7%)	14.0%	800,899	105,385 (13.2%)	< 0.001
Prediabetes (Y)	776,764	29,232 (3.8%)	24,135	1588 (6.6%)	2.8%	800,899	30,820 (3.8%)	< 0.001
Anaemia (Y)	776,764	60,875 (7.8%)	24,135	3160 (13.1%)	5.3%	800,899	64,035 (8%)	< 0.001
Ischaemic Cardiomyopathy (Y)	776,764	18,812 (2.4%)	24,135	2134 (8.8%)	6.4%	800,899	20,946 (2.6%)	< 0.001
Dyslipidaemia (Y)	776,764	250,256 (32.2%)	24,135	11,039 (45.7%)	13.5%	800,899	261,295 (32.6%)	< 0.001
AF (Y)	776,764	24,423 (3.1%)	24,135	2852 (11.8%)	8.7%	800,899	27,275 (3.4%)	< 0.001
Hypertension (Y)	776,764	262,190 (33.8%)	24,135	14,256 (59.1%)	25.3%	800,899	276,446 (34.5%)	< 0.001
Chronic kidney failure (Y)	776,764	29,259 (3.8%)	24,135	2823 (11.7%)	7.9%	800,899	32,082 (4%)	< 0.001
Heart Failure (Y)	776,764	13,253 (1.7%)	24,135	2672 (11.1%)	9.4%	800,899	15,925 (2%)	< 0.001
Stroke (Y)	776,764	22,330 (2.9%)	24,135	1944 (8.1%)	5.2%	800,899	24,274 (3%)	< 0.001
Lung Cancer (Y)	776,764	1740 (0.2%)	24,135	552 (2.3%)	2.1%	800,899	2292 (0.3%)	< 0.001
Fibrinogen	59,544	375.2 ± 94.3	3556	407.2 ± 105.7	32.01	63,100	377 ± 95.3	< 0.001
C-reactive protein	56,406	8.3 ± 20.9	3367	15.3 ± 32.5	7.05	59,773	8.7 ± 21.7	< 0.001

Age is strongly associated to COPD in both genders, but the effect is higher in men. Moderate and high alcohol consumption in men represents 1.3 risk of COPD (*p* < 0.001). A history of smoking (smoker or ex-smoker) multiplies this risk by 3.5 (*p* < 0.01). Men with bronchiectasis presented a risk 20.5 times higher of suffering from COPD (*p* < 0.001). Other frequent comorbidities in patients with COPD were anaemia (aOR = 1.2; *p* < 0.001) and ischaemic cardiomyopathy (aOR = 1.2; *p* < 0.001). Cor pulmonale and heart failure were also more prevalent amongst COPD patients (aOR = 5.3; *p* < 0.001 and aOR = 2.4; *p* < 0.001, respectively). Table 3 describes the crude and adjusted effect of comorbidities in men.

**Table 3:** Univariate and multivariate analysis of the study variables in men

Men	Descriptive		Crude effect			Adjusted effect		
	N	n (%)	OR	CI (95%)	*p*	OR	CI (95%)	*p*
Age grouped	389,030							
41—50		134,104 (34.5%)	1	–		1	**–**	
51—60		97,416 (25%)	4.4	(4.1—4.8)	< 0.001	3.4	(3.1—3.7)	< 0.001
61—70		76,965 (19.8%)	13.2	(12.2—14.3)	< 0.001	8.7	(8—9.4)	< 0.001
71—80		51,763 (13.3%)	27.1	(25.1—29.3)	< 0.001	18.8	(17.3—20.5)	< 0.001
80 or more		28,782 (7.4%)	34.2	(31.5—37.1)	< 0.001	29.1	(26.6—31.8)	< 0.001
Smoking	389,030	173,001 (44.5%)	4.1	(4—4.3)	< 0.001	3.5	(3.4—3.6)	< 0.001
Bronchiectasis	389,030	10,479 (2.7%)	31.3	(30—32.7)	< 0.001	20.5	(19.5—21.6)	< 0.001
Cor Pulmonale	389,030	451 (0.1%)	21.0	(17.4—25.2)	< 0.001	5.3	(4.3—6.7)	< 0.001
Lung Cancer	389,030	1790 (0.5%)	8.2	(7.4—9.1)	< 0.001	3.6	(3.2—4)	< 0.001
Heart Failure	389,030	7155 (1.8%)	7.9	(7.4—8.3)	< 0.001	2.4	(2.3—2.6)	< 0.001
OSAS	389,030	8767 (2.3%)	2.9	(2.7—3.1)	< 0.001	1.7	(1.6—1.9)	< 0.001
Osteopathy	389,030	3228 (0.8%)	4.4	(4.1—4.9)	< 0.001	1.6	(1.5—1.8)	< 0.001
Alcohol Consumption Risk Rate	389,030							
No intake		94,120 (24.2%)	1		< 0.001	1		< 0.001
Low		80,222 (20.6%)	0.9	(0.9—0.9)	< 0.001	0.9	(0.9—1)	< 0.001
Moderate or High		7855 (2%)	1.1	(1—1.2)	0.006	1.3	(1.1—1.4)	< 0.001
NA		206,833 (53.2%)	0.3	(0.3—0.3)	< 0.001	0.7	(0.7—0.8)	< 0.001
Depression	389,030	30,568 (7.9%)	1.4	(1.4—1.5)	< 0.001	1.3	(1.2—1.3)	< 0.001
Ischaemic Cardiomyopathy	389,030	14,546 (3.7%)	3.1	(2.9—3.3)	< 0.001	1.2	(1.1—1.3)	< 0.001
AF	389,030	13,974 (3.6%)	4.1	(3.9—4.3)	< 0.001	1.2	(1.1—1.3)	< 0.001
Anaemia	389,030	17,209 (4.4%)	3.3	(3.2—3.5)	< 0.001	1.2	(1.1—1.2)	< 0.001
Chronic kidney failure	389,030	16,268 (4.2%)	3.5	(3.3—3.6)	< 0.001	1.1	(1—1.2)	0.001
Dyslipidaemia	389,030	122,087 (31.4%)	1.9	(1.8—1.9)	< 0.001	1.0	(0.9—1)	0.009
Hypertension	389,030	131,174 (33.7%)	2.9	(2.8—3)	< 0.001	0.9	(0.9—1)	0.004
DM2	389,030	58,219 (15%)	2.3	(2.3—2.4)	< 0.001	0.9	(0.9—1)	< 0.001
Grouped BMI	389,030							
Normal		25,352 (6.5%)	1	–		1	–	< 0.001
Overweight		67,457 (17.3%)	0.8	(0.7—0.8)	< 0.001	0.8	(0.7—0.8)	< 0.001
Obesity		47,140 (12.1%)	0.9	(0.8—0.9)	< 0.001	0.9	(0.8—0.9)	< 0.001
NA		249,081 (64%)	0.2	(0.2—0.2)	< 0.001	0.7	(0.7—0.8)	< 0.001
ROC Curve		0.896 (0.89—0.9)						

In women, the risk to develop COPD between 61 and 70 years of age increased by 4.7, and by 14.5 from 80 years onwards (*p* < 0.001). While low alcohol consumption did not increase the risk to develop COPD, (aOR = 0.9; *p* = 0.22), moderate to high drinking increased the risk by 1.6 (*p* < 0.001). Bronchiectasis increased by 27.1 the risk to develop COPD (*p* < 0.001). History of smoking increased almost five fold the risk to develop COPD (aOR = 4.7; *p* < 0.001). In women with COPD, comorbidities such as cor pulmonale and history of lung cancer were 3 times higher (aOR = 3.1; *p* < 0.001and aOR = 3.6; *p* < 0.001, respectively). Table 4 shows the remaining comorbidities.

**Table 4:** Univariate and multivariate analysis of the study variables in women

Women	Descriptive		Crude effect			Adjusted effect		
	N	n (%)	OR	CI (95%)	*p*	OR	CI (95%)	*p*
Age grouped	411,869							
41–50		120,969 (29.4%)	1	–		1	–	
51–60		97,887 (23.8%)	2.8	(2.5–3.1)	< 0.001	2.5	(2.2–2.8)	< 0.001
61–70		81,798 (19.9%)	4.2	(3.8–4.7)	< 0.001	4.7	(4.1–5.3)	< 0.001
71–80		61,424 (14.9%)	7.1	(6.3–7.9)	< 0.001	8.9	(7.8–10.1)	< 0.001
80 or more		49,791 (12.1%)	11.0	(9.8–12.2)	< 0.001	14.5	(12.7–16.6)	< 0.001
Smoking	411,869	89,687 (21.8%)	2.4	(2.3–2.5)	< 0.001	4.7	(4.4–5)	< 0.001
Lung Cancer	411,869	502 (0.1%)	5.9	(4.2–8.2)	< 0.001	3.6	(2.5–5.2)	< 0.001
Bronchiectasis	411,869	4994 (1.2%)	32.6	(30.4–35)	< 0.001	27.1	(25–29.3)	< 0.001
Lung Cancer	411,869	502 (0.1%)	5.9	(4.2–8.2)	< 0.001	3.6	(2.5–5.2)	< 0.001
Cor Pulmonale	411,869	505 (0.1%)	13.2	(10.3–16.9)	< 0.001	3.1	(2.3–4.1)	< 0.001
Heart Failure	411,869	8770 (2.1%)	8.2	(7.6–8.9)	< 0.001	3.0	(2.7–3.3)	< 0.001
OSAS	411,869	2884 (0.7%)	4.4	(3.7–5.2)	< 0.001	2.2	(1.8–2.6)	< 0.001
Alcohol Consumption Risk Rate	411,869							
No intake		172,572 (41.9%)	1		< 0.001	1		< 0.001
Low		33,322 (8.1%)	0.9	(0.8–1)	0.002	0.9	(0.9–1)	0.220
Moderate or High		1277 (0.3%)	1.9	(1.4–2.5)	< 0.001	1.6	(1.2–2.3)	0.005
NA		204,698 (49.7%)	0.4	(0.4–0.4)	< 0.001	0.8	(0.7–0.8)	< 0.001
Ischaemic Cardiomyopathy (no AMI)	411,869	6400 (1.6%)	3.7	(3.3–4.1)	< 0.001	1.3	(1.1–1.5)	< 0.001
Osteopathy	411,869	46,798 (11.4%)	2.2	(2.1–2.4)	< 0.001	1.3	(1.2–1.4)	< 0.001
Depression	411,869	76,067 (18.5%)	1.6	(1.5–1.7)	< 0.001	1.3	(1.2–1.3)	< 0.001
Hypertension	411,869	145,272 (35.3%)	3.0	(2.8–3.1)	< 0.001	1.2	(1.1–1.3)	< 0.001
DM2	411,869	47,166 (11.5%)	2.3	(2.1–2.4)	< 0.001	1.1	(1–1.2)	0.008
Anaemia	411,869	46,826 (11.4%)	1.5	(1.4–1.6)	< 0.001	1.1	(1–1.2)	0.018
FA	411,869	13,301 (3.2%)	4.0	(3.7–4.3)	< 0.001	1.1	(1–1.3)	0.014
Chronic kidney failure	411,869	15,814 (3.8%)	3.0	(2.8–3.3)	< 0.001	1.1	(1–1.2)	0.108
Dyslipidaemia	411,869	139,208 (33.8%)	1.8	(1.7–1.9)	< 0.001	1.0	(0.9–1)	0.336
Grouped BMI	411,869							
Normal		37,589 (9.1%)	1	–		1.0	–	
Overweight		59,722 (14.5%)	0.9	(0.8–1)	0.003	0.8	(0.7–0.9)	< 0.001
Obesity		59,011 (14.3%)	1.2	(1.1–1.3)	< 0.001	1.0	(0.9–1.1)	0.461
NA		255,547 (62%)	0.4	(0.4–0.5)	< 0.001	0.9	(0.8–1)	0.099
ROC Curve		0.863 (0.86–0.87)						

## Discussion

The prevalence of COPD in the 800,899 population was 3%. This prevalence is lower than that published in the EPISCAN (10.2%) and IBERPOC (9.1%) studies [3]. It is also lower than the prevalence indicated in other studies conducted in the Spanish population [16, 18], which showed COPD prevalences around 9%. The 3% prevalence obtained in this study might respond to under-reporting in the primary care setting caused by mild symptomatology (most patients would be classified as GOLD stages1-2, with mMRC < 2 and CAT < 10) and under-recording of spirometry results. Experts believe that a significant number of patients remain undiagnosed [6]. The higher proportion of men than women (77.1% vs 22.9%, respectively) diagnosed with COPD is corroborated. However, the impact of shared risk factors such as age, smoking exposure and alcohol consumption appears to be greater in women.

As previously reported [9, 19], comorbidities are common in patients with COPD (prediabetes and diabetes, ischaemic cardiomyopathy, dyslipidaemia, hypertension, heart failure, atrial fibrillation, history of stroke, lung cancer and chronic kidney disease). Mild reductions in airway flux in patients with COPD have been associated with higher risk of stroke, ischaemic cardiomyopathy and sudden cardiac death. It has been suggested that COPD exacerbations generate inflammatory processes which increase C-reactive protein, fibrinogen and endothelin-1 vasoconstrictor peptide in peripheral blood. These factors participate in the genesis of atherosclerosis [20], the decline in vascular function and endothelial dysfunction, causing cardiovascular complications. Another study conducted in a Spanish population found similar prevalences of hypertension (43%), dyslipidaemia (33%) and diabetes (16%) [21]. Additionally, 13.3% of COPD patients in our study suffered from anaemia, similarly to other series reporting percentages over 10% [22, 23]. Anaemia reduces exercise capacity. While the pathophysiology of anaemia in COPD is still debated, some authors have suggested that the inflammation of COPD mediated by INF-a, IL-1, IL-6 and IFN-gamma induces changes in iron metabolism, reducing the intestinal absorption of this metal and consequently affecting haematopoiesis [24]. Studies show an increase in the proportion of anaemia in older and more severe (GOLD) cases of COPD. For instance, Watz et al. reported a 14% prevalence of anaemia in patients with very severe COPD (GOLD 4) [25]. Since most COPD patients have a long history of smoking, lung cancer is more frequent in this group of patients. One study showed that cancer was the cause of death in 16.1% of patients with COPD, with lung cancer being the most common [26]. As previously described, bronchiectasis are a significant risk factor of COPD [27]

COPD is more prevalent in older men. Prevalence is higher in men between 71 and 80 and over 80 years of age (ORa = 18.8, *p* < 0.001 and ORa = 29.1, *p* < 0.001), respectively. Some research indicates that irreversible bronchial obstruction and decline of the FEV1 increase with age [28]. Another risk factor is moderate-high alcohol consumption, which is 1.3 higher in men with a diagnosis of COPD compared to those not diagnosed with this condition. Interestingly, in men with a diagnosis of COPD, moderate to high alcohol consumption is associated with higher use of tobacco [19]. In this respect, the association between smoking and COPD [7] has long been recognized, and men with a history of smoking have are 3.5 times more likely to have COPD. The most frequent comorbidities in men with COPD are cor pulmonale (5.3 times more prevalent in men with than without COPD) and heart failure (2.4 times more common in males with COPD). Our study has shown a lower ORa than the study by Villar et al., which reported an ORa of 4.5 of heart failure in patients with COPD [29]. Pathophysiologically, the use of systemic corticosteroids to treat exacerbations in moderate and severe COPD can generate fluid retention and over time, this increase in blood volume might cause heart failure [20]. Additionally, treatment with beta-adrenergic bronchodilators might cause tachycardia, an increase in myocardial oxygen consumption and thus aggravation of symptoms in patients with heart failure [30]. Anaemia was also 1.2 times more frequent in patients with COPD.

Older age is also a risk factor for COPD in women, and prevalence is 9 times higher in women between 71 and 80 years of age (ORa = 8.8; *p* < 0.001). Women with moderate to high alcohol consumption are at higher risk than men of COPD (ORa = 1.6 vs. ORa = 1.3, respectively). Similarly, the risk in women with a history of smoking is also higher than in men (risk 4.7 vs. 3.5 times higher to present COPD, respectively). Some authors have suggested that the higher risk of women to develop COPD can be explained by their higher susceptibility to the effects of smoking, their longer exposure to indoor pollution, and by anatomic and hormonal characteristics [12, 31]. The risk of presenting bronchiectasis was higher in women with COPD (ORa = 27.1) than in their male counterparts (ORa = 20.5). This finding has also been described by Oliveira et al. [27], which analysed a cohort of 20,047 patients with bronchiectasis from 36 health centres in Spain, and concluded that 54.9% were women. The most common causes for bronchiectasis were systemic, idiopathic and post-infectious diseases. Other comorbidities such as obstructive sleep apnoea syndrome (OSAS), are also more common in women than in men (twofold increase in prevalence in women with COPD, compared to 1.7 in men with COPD). The ORc for diabetes was 2.3 for male and female COPD patients. This risk is attributed to the hyperglycaemia caused by the treatment of moderate and severe COPD exacerbations with systemic corticosteroids, which over time might cause diabetes in a subgroup of COPD patients [32]. However, this risk decreased in the multivariate analysis.

### Limitations

Since the information has been obtained from the SIDIAP database (data that originate from the electronic medical records), under-recording should be considered. An additional limitation to consider is the fact that no spirometric curves of patients included in the study have been analysed. Also, in a prevalence study the associations might not be causal. For instance, comorbidities such as bronchiectasis can be risk factors for COPD, whereas associated diseases such as cor pulmonale could be the result of this condition. Furthermore, the large population sample might produce statistically significant differences with no clinical relevance. The classification of the database follows the algorithm proposed by Barrecheguren et al. [15], and thus false positives and false negatives need to be considered. Another limitation when working with a large database would be under-reporting some data from the medical records of patients, as the FEV1/FVC case. In order to minize this fact, Barrecheguren et al. [15] algorithm was used in order to be able to include higher number of patients and to ensure high statistical power of the study.

## Conclusion

COPD prevalence in the SIDIAP cohort is lower than expected (3%), probably due to under-recording of clinical information in primary care medical records. Patients suffering from COPD present high comorbidity rates. Although in general risk factors are the same in both genders, women appear to be more susceptible to harmful exposures such as smoking and present a higher proportion of bronchiectasis and OSAS. Understanding associated comorbidities is crucial for an integral approach to COPD. Further research in similar cohorts would be required to provide new evidence on the prevalence of COPD as well to determine the incidence of disease exacerbation based on clinical-epidemiological features.

## Availability and data and materials

The datasets used and/or analysed during the current study are available from the corresponding author on reasonable request.
